# Active metabolites combination therapies: towards the next paradigm for more efficient and more scientific Chinese medicine

**DOI:** 10.3389/fphar.2024.1392196

**Published:** 2024-04-18

**Authors:** Quan Gao, Hao Wu, Min Chen, Xidong Gu, Qibiao Wu, Tian Xie, Xinbing Sui

**Affiliations:** ^1^ State Key Laboratory of Quality Research in Chinese Medicines, Faculty of Chinese Medicine, Macau University of Science and Technology, Taipa, China; ^2^ College of Pharmacy, Hangzhou Normal University, Hangzhou, China; ^3^ Department of Breast Surgery, The First Affiliated Hospital of Zhejiang Chinese Medical University, Hangzhou, China

**Keywords:** TCM formulae, active metabolites, combination therapies, multi-omics, single cell analysis

## Abstract

Traditional Chinese medicine (TCM) formulae have been studied extensively in various human diseases and have proven to be effective due to their multi-component, multi-target advantage. However, its active metabolites are not clear and the specific mechanisms are not well established, which limits its scientific application. Recently, combination therapies are attracting increasing attention from the scientific community in the past few years and are considered as the next paradigm in drug discovery. Here, we tried to define a new concept of “active metabolites combination therapies (AMCT)” rules to elucidate how the bioactive metabolites from TCMs to produce their synergistic effects in this review. The AMCT rules integrate multidisciplinary technologies like molecular biology, biochemistry, pharmacology, analytical chemistry and pharmacodynamics, etc. Meanwhile, emerging technologies such as multi-omics combined analysis, network analysis, artificial intelligence conduce to better elucidate the mechanisms of these combination therapies in disease treatment, which provides new insights for the development of novel active metabolites combination drugs. AMCT rules will hopefully further guide the development of novel combination drugs that will promote the modernization and international needs of TCM.

## 1 Introduction

The traditional Chinese medicine (TCM) prescription has been used in clinical practice for thousands of years in China. TCM formulae treat complicated diseases through combined botanical species or minerals which follow specific compatibility principles “Jun-Chen-Zuo-Shi” (Luan et al., 2020). However, due to the deficiency of traditional research methods, the scientific theoretical basis of TCM compatibility and the specific active metabolites are still unclear. Also, the quality control standards of TCM preparations are not well established and the therapeutic effect of some botanical drugs is not satisfied (Wang et al., 2020). Moreover, compatibility in English means “the ability of people or things to live or exist together without problems” or “the ability of machines, especially computers, and computer programs to be used together.” It can not represent the principles “Jun-Chen-Zuo-Shi” and it is also difficult for foreigners to understand. Therefore, it is urgent to develop novel Chinese drugs to improve the efficacy and clarify their pharmacological effects in modern scientific language based on the combinations of active metabolites in TCM formulae.

Recently, combination therapies (in which two or more drugs are used to target two or more pathways) are attracting increasing attention from the scientific community in the past few years, and were significantly more effective compared to monotherapies (Plana et al., 2022). For example, S-1, an oral combination pills of tegafur (FF) and two types of enzyme inhibitor 5-chloro-2,4-dihydroxypyridine (CDHP) and potassium oxonate (Oxo) in a molar ratio of 1:0.4:1, was demonstrated to be a useful alternative to 5-FU for cancer patients (Nakamura et al., 2006). CaDUET, a combination of amlodipine besylate and atorvastatin calcium in different dose ratios, was approved by the US Food and Drug Administration (FDA) for the treatment of hypertension and hypercholesterolemia (Dennison et al., 2017). Sulperazon, a combination injection of cefoperazone and sulbactam in a molar ratio of 2:1, was proved to be a broad-spectrum antibiotic that can act against a wide range of microorganisms (Lai et al., 2018).

Here, an “active metabolites combination therapies (AMCT)” concept will be proposed by considering the deficiencies present in TCM formulae and the advantages of combination therapies, to settle the issues in active metabolites, molecular mechanisms, and clinical efficacy. Different from the traditional “Jun-Chen-Zuo-Shi” compatibility, AMCT possesses clear drug characteristics and targets, and aligns with the fundamental principles of TCM. Active ingredients-based combination therapies are thus considered more scientific and beneficial to accelerating the modernization and international needs of TCM. This review provides a detailed description of the definition and ideas for the new concept of AMCT and summarizes on the application of emerging technologies like single-cell multiomics to elucidate the molecular mechanisms of AMCT drug, which will hopefully promote the development and international needs of TCM.

## 2 Concept of AMCT

AMCT differs from decoction-ready medicines compatibility, component compatibility as well as molecular compatibility (Table 1) which refers to analyzing the active metabolites of clinically effective TCM formulae by modern technologies, determining the combinations ratio of these active metabolites according to the best dose-response relationship, and then elucidate the molecular mechanisms of the novel Chinese medicine in modern scientific language. From the definition, the ideas for the modernization of AMCT can be outlined in four aspects: 1) The new Chinese medicines’ active metabolites are derived from clinically effective TCM formulae rooted in TCM-based theory and clinical practicality. 2) The application of modern technology to analyze active metabolites, determine combination ratios, and elucidate mechanisms of action aligns with Western medicine principles, which enhances the applicability of Chinese medicine in personalized treatment and promotes the internationalization of Chinese medicine. 3) The objective of this novel combination drug is to surpass or equal the effectiveness of the original TCM prescription, thereby supporting the modernization of Chinese medicine. 4) The AMCT rules emphasize the significance of exploring the synergistic effects of combining multiple active ingredients from clinically effective TCM formulae. Unlike traditional studies that mainly focus on screening and evaluating single active metabolites, the AMCT rules address the limitations of conventional approaches, such as the vague understanding of the pharmacodynamic substances in TCM formulae and the narrow focus on the effects of individual components in Western medicine.

**TABLE 1:** Comparison of active metabolites combination therapy preparations with other compatibility preparations.

	Decoction-ready medicines compatibility preparations	Component compatibility preparations	Molecular compatibility preparations	Active metabolites combination therapy preparations
Theoretical basis	Traditional theories such as “monarch, minister, assistant, envoy,” “seven compatibility methods,” and so on	Modern pharmaceutical theories	Modern pharmaceutical theories and molecular biologic theories	Modern pharmaceutical theories and molecular biologic theories
Prescription composition	Many constituents and very complex	Many constituents and very complex	Fewer constituents and relatively simpler	Simple
Active ingredients	Various blends, mostly unclear	Coarse extracts, partially clear	Active ingredient, basically clear	Active metabolite, clear
Compatibility type	Decoction piece and decoction piece compatibility	Component and component compatibility	Natural ingredients with homologues structural types compatibility from one herb	Compatibility of different active metabolites with the best molar ratio from the same TCM formulae
Action mechanism	Basically unclear	Partially clear	Basically clear	Clear
Preparation features	Traditional preparations (decoction, pills, powders, ointments, and so on)	Standard preparations (capsules, tablets, granules, and so on)	Modern preparations (such as nano preparations, and so on)	Any dosage form
Quality control	Less quality control	Some quality control	Stable and controlled quality	More stable and controlled quality

Altogether, AMCT rules follow the thinking “clinical experience-basic research-clinical application” to establish a paradigm for developing more efficient and more scientific Chinese medicine.

## 3 Approaches for establishing AMCT

**FIGURE 1:**
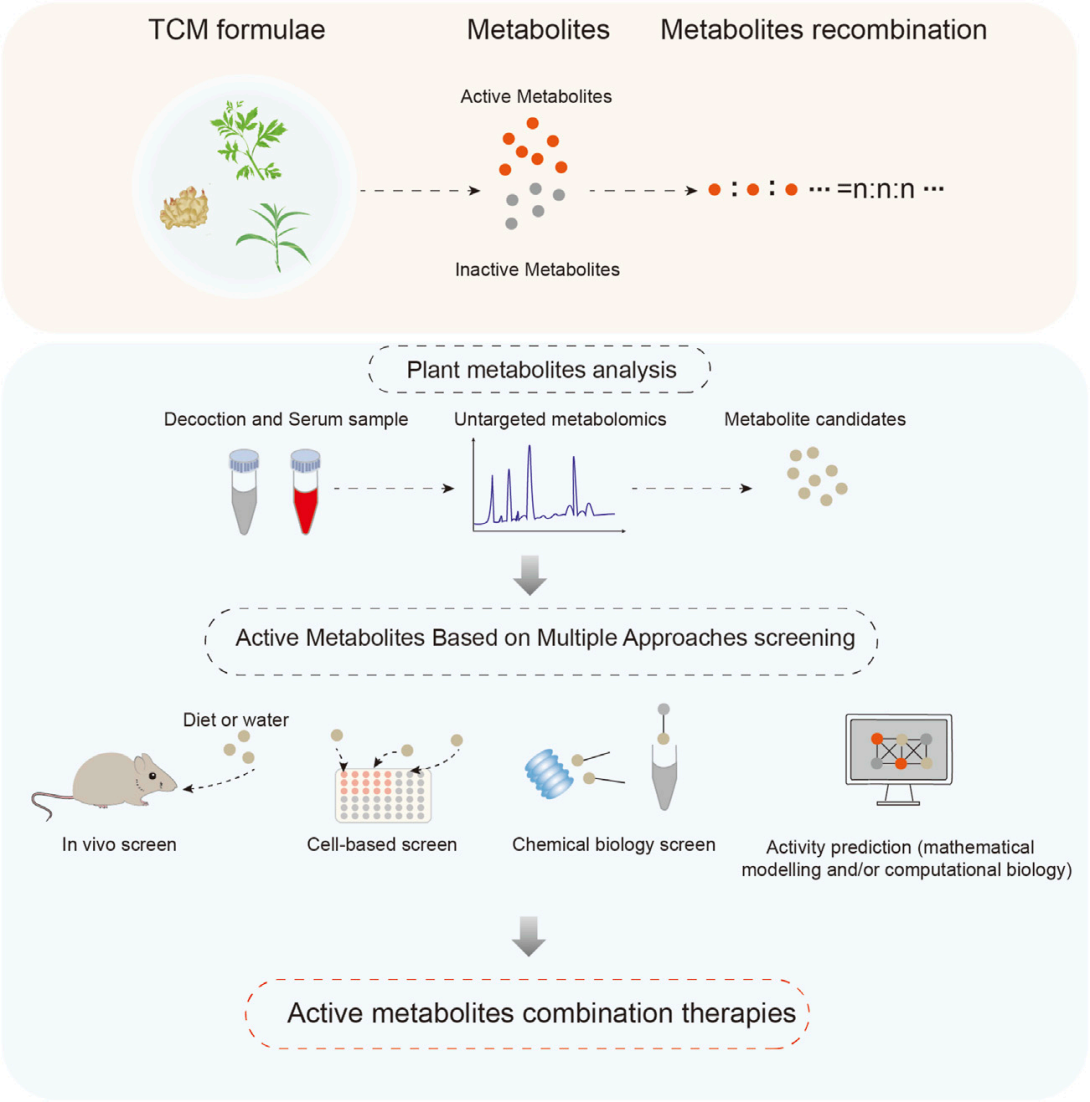
Active metabolites combination therapies (AMCT) aims to analyzing the active metabolites of clinically effective TCM formulae by modern technologies, determining the combinations ratio of these active metabolites according to the best dose-response relationship, and then elucidate the molecular mechanisms of the novel Chinese medicine, and finally used in clinical treatment.

TCM formulae have been proven effective in clinical treatment and serve as a valuable resource for novel drug development from TCM (Cheung, 2011; Ma et al., 2013). Currently, Chinese materia medica preparation, such as Lianhua Qingwen capsule (Shen and Yin, 2021), and Jingfang Granules (Xu Q. et al., 2023), are directly or indirectly derived from classic TCM formulae, but limitations remain. Drugs of controlled quality and clear mechanisms facilitate clinical monitoring of drug metabolism and rational prevention of adverse reactions. Therefore, combining strategies for active metabolites analysis and mechanism research, a comprehensive research strategy of AMCT was proposed and established. This strategy consists of four key steps: identifying formulation metabolites, identifying active metabolites, optimizing component synergy, and evaluating the mechanism and efficacy of combination drugs (Figure 1).

### 3.1 Identifying formulation metabolites

Formulation metabolites analysis is the process of determining the pharmacodynamic material basis in a TCM formula. Notably, the pharmacodynamic material basis of formulae is defined as the multiple chemical components in a formula that exert therapeutic effects on specific diseases (Zhang et al., 2020). Currently, various strategies have been developed to identify the metabolites, including serum pharmacology methods, mass spectrometry imaging technology, etc.

The serum pharmacology methods employ contemporary separation methodologies to analyze the constituents of blood post-oral administration of TCM formulations in humans or animals, with the objective of identifying metabolites within the serum. This process includes the preparation and quality control of TCM, administration of the medicine, blood collection and serum preparation, as well as the analysis and detection of metabolites (Feng et al., 2020). Xu et al. (Xu H. et al., 2023) elucidated the pharmacological material basis of Huashi Baidu decoction, consisting of *Ephedra sinica Stapf* (*Ephedraceae*; *Ephedrae Herba*), *Pogostemon cablin (Blanco) Benth.* (*Lamiaceae*; *Pogostemonis Herba*), CaSO₄·2H₂O, *Magnolia officinalis Rehder & E.H.Wilson* (*Magnoliaceae*; *Magnoliae Officinalis Cortex*), *Astragalus mongholicus Bunge* (*Fabaceae*; *Astragali Radix*), *Atractylodes lancea (Thunb.) DC.* (*Asteraceae*; *Atractylodis Rhizama*), *Amomum tsao-ko Crevost & Lemarié* (*Zingiberaceae*; *A. tsao-ko Fructus Maturus*), *Pinellia ternata (Thunb.) Makino* (*Araceae*, *Pinellia Rhizoma*), *Descurainia sophia (L.) Webb ex Prantl* (*Brassicaceae*; *Descurainia Semen, Lepidii Semen*), *Paeonia anomala L. (Paeoniaceae*; *Paeoniae Radix Rubra), Prunus armeniaca L.* (*Rosaceae*; *Semen Armeniacae Amarum Maturus*), *Poria cocos (Schw.) Wolf* (*Polyporaceae*; *Poriae*), *Glycyrrhiza glabra L.* (*Leguminosae; Glycyrrhizae Radix et Rhizoma*), *Rheum palmatum L.* (*Polygonaceae*; *Rhei Radix et Rhizoma*), using animal model extraction of drug-containing serum combined with UPLC-Q-TOF/MS. The results showed that 343 metabolites were identified *in vitro* and 60 prototype metabolites were traced *in vivo*. However, this technique has its limitations, such as variations in metabolite concentration, blood collection volume, timing of blood collection, and individual differences, all of which can lead to discrepancies in the metabolites identified.

Mass spectrometry imaging (MSI) is an advanced technology that combines classical mass spectrometry and ion imaging. It is capable of visualizing various types of metabolites of TCM and accurately describing the biosynthesis and characteristic spatial distributions of functional metabolites. This is an effective method for in-depth analysis and understanding of TCM composition and metabolite biosynthesis (Huang et al., 2022). Guo et al., 2023 utilized AFADESI-MSI to detect the metabolites in *Pueraria montana (Lour.) Merr.* (*Fabaceae*; *Puerariae Lobatae Radix*), as well as the distribution of these metabolites in micro-regions. Moreover, Li et al., 2021 used MALDI-MSI to characterize the metabolites and their distribution in *P. anomala L. (Paeoniaceae*; *Paeoniae Radix Alba)*.

### 3.2 Identifying active metabolites

#### 3.2.1 Pharmacologically based screening strategies for active metabolites

The strategies of traditional pharmacology for identifying active metabolites include: First, by employing a formulation metabolites analysis strategy, the metabolites of TCM formulae are extracted, separated, and identified. Next, various biological methods are used, along with pharmacological models (2D cellular models, 3D organoid models, animal models, etc.) to screen the biological activity of each metabolite. Ultimately, it determines which metabolites are active metabolites. The Huashi Baidu decoction is an approved formula for COVID-19 treatment that has antiviral and anti-inflammatory properties (Liu et al., 2021; Xiong et al., 2022). Xu et al. (Xu H. et al., 2023) employed Ultrahigh-performance liquid chromatography with quadrupole time-of-flight mass spectrometry (UPLC-Q-TOF/MS) in conjunction with serum pharmacology strategies, successfully identifying 60 prototype metabolites. Following that viral and inflammatory cell models are utilized for the identification of active metabolites, with prototype compounds that demonstrate an inhibition rate exceeding 90% after drug treatment being defined as active metabolites. The results showed that magnolol, glycyrrhisoflavone, licoisoflavone A, emodin, echinatin, and quercetin exhibited significant antiviral activity, while licochalcone B, echinatin, and glycyrrhisoflavone demonstrated anti-inflammatory effects.

#### 3.2.2 Disease target-based screening strategies for active metabolites

Disease target-based active ingredient screening is an important strategy for active metabolite screening that relies primarily on a thorough understanding of the disease mechanism and the precise identification and validation of disease-related targets. It is characterized by high efficiency, specificity, and efficacy compared to traditional drug screening. This strategy primarily integrates chemistry, biotechnology, and computer-aided drug design technology, etc.

The chemical and biological techniques for screening active metabolites encompass affinity ultrafiltration mass spectrometry, and surface plasmon resonance technology (SPR), among others. Affinity ultrafiltration mass spectrometry is an analytical technique that combines affinity ultrafiltration with mass spectrometry (Wei et al., 2016). This technology utilizes affinity ultrafiltration to separate and enrich active metabolites of TCM that have a strong affinity to specific targets, followed by identification and analysis through mass spectrometry. Zhu et al., 2022 established an analytical method based on affinity ultrafiltration coupled with UHPLC-Q-Exactive Orbitrap mass spectrometry for the rapid screening and identification of active metabolites targeting estrogen receptors (ERα and ERβ). From *Arnebiae guttata Bunge* (*Boraginaceae*; *Arnebiae Radix*), they identified 14 potential active metabolites binding to ERα and 10 potential active metabolites binding to ERβ. Moreover, SPR technology, due to its ability to monitor biomolecular interactions directly without labeling, has become a key tool in screening for active metabolites in TCM. This technology allows for the rapid and accurate identification of metabolites that bind to specific targets from formulae (Olaru et al., 2015). Lv et al., 2022, focusing on TNF-R1 as a critical anti-inflammatory target, utilized SPR technology to identify active metabolites against TNF-R1 from *Paeonia lactiflora Pall. (Paeoniaceae*; *Paeoniae Radix Alba)*, successfully determining paeoniflorin and paeonol as two primary active metabolites.

Computer-aided drug design technology used for identifying active ingredients encompasses molecular docking (Pinzi and Rastelli, 2019) and pharmacophore modeling (Lu et al., 2018), among others. These two approaches complement each other: molecular docking precisely predicts the binding mode and affinity between a compound and its target by simulating their direct interaction, while pharmacophore modeling focuses on identifying the core features responsible for a compound’s activity. Integrating these strategies allows for the efficient screening and identification of potential active metabolites. Molecular docking and pharmacophore modeling have their value in predicting the interactions between drugs and targets, but they are limited in accuracy, such as the potential for nonspecific binding. Therefore, these methods can serve as preliminary means for screening active metabolites. However, to obtain more scientifically reliable theoretical support, it is necessary to validate the findings through a series of experimental methods, including SPR, thermal stability analysis, and other techniques, to ensure the accuracy and reliability of the results.

### 3.3 Optimizing component synergy

Traditional research methods have not considered the TCM formulae as a whole system, but rather selected single active metabolites for study, neglecting the interactions between active metabolites and their close connection with the organism. Therefore, we further propose the combinations of active metabolites to achieve their efficacy greater than or equal to the efficacy of the original formulae. The assessment of drug synergy often involves pharmacological evaluations (*in vivo* and *in vitro* experiments) and mathematical model predictions (Random Forests, Support Vector Machines (SVM), Neural Networks, and Deep Learning models). These methods can forecast whether there is synergy between active metabolites and optimize combination ratios. For instance, the use of synergy index (CI) (Long et al., 2015) and dose-effect curves helps evaluate whether drugs have synergistic, additive, or antagonistic effects. These techniques aid in discovering synergistic drug combinations and optimizing drug combination ratios to enhance therapeutic effects and reduce side effects. Notably, the optimum combination ratio of the active metabolite combination (biagents, triple combination, etc.) is optimized by considering the content ratio of the original formula, leading to the attainment of the most effective therapeutic outcome. She et al., 2022 developed DeepMDS, a deep learning-based model for predicting drug combinations that may have synergistic effects in specific cell lines. The team selected seven FDA-approved breast cancer therapeutics to predict synergistic combinations using the DeepMDS model and validated the predictions with pharmacological evaluation and CIs. In addition, the model is also suitable for predicting synergistic combinations of active metabolites in TCM, providing a new tool for understanding drug interactions and optimizing therapeutic strategies. However, the application of deep learning in predicting drug combinations is constrained by factors such as sparse data, the complexity of drug interactions, and computational resource requirements. These limitations compromise the accuracy and practicality of the models.

### 3.4 Elucidating synergistic mechanisms

Studying novel combined drug mechanisms has important theoretical and usage implications in the fields of pharmacy and medicine. Meanwhile, understanding these mechanisms will also enhance drug adaptability to treat diseases effectively while minimizing side effects. The synergistic effects of multi-metabolite drugs are categorized into those targeting a single site and those affecting multiple sites. Currently, methodologies such as multi-omics combined analysis, network analysis, artificial intelligence, machine learning and computational biology are employed to study these effects. These strategies offer new perspectives and tools for unraveling the synergistic mechanisms of combination drugs.

Multi-omics combined analysis technology is considered cutting-edge in the field of life science, offering insights into the complex treatment mechanisms of combination therapies. This advanced technology enables researchers to explore disease treatment mechanisms at various levels, including the genome, transcriptome, proteome, and metabolome (Lee et al., 2020). The advancement of multi-omics combined analysis technology has broadened its applications in drug research, including drug screening, efficacy evaluation, pharmacology, and related areas. Zhang et al., 2021 utilized scRNA-seq, TCR-seq, and ATAC-seq to analyze immune cell dynamics of tumor microenvironment and peripheral blood of patients with triple-negative breast cancer (TNBC) undergoing treatment with either paclitaxel alone or paclitaxel plus atezolizumab, and found that the paclitaxel regimen decreased levels of CD8-CXCL13, CD4-CXCL13, Tregs, and Bfoc cells, while levels increased in the paclitaxel plus atezolizumab combination. This differential response implies that the paclitaxel regimen can target key anti-tumor immune cells in TNBC, concurrently promoting immunosuppressor macrophages. Consequently, the decrease in these immune cells induced by paclitaxel therapy may compromise the therapeutic efficacy of atezolizumab in TNBC, shedding new light on combining paclitaxel and atezolizumab for TNBC treatment. *Boswellia ameero Balf.f.* (*Burseraceae*; *Olibanum*) and *Commiphora myrrha (Nees) Engl.* (*Burseraceae*; *C. myrrha Resina*) have been used in the treatment of cerebrovascular disease (Liao et al., 2021), with 11-keto-β-boswellic acid (KBA) and Z-Guggulsterone (Z-GS) being the main metabolites (Ding et al., 2015; Liu et al., 2022). In the study conducted by Liu et al., 2023, the investigators explored the synergistic effects and underlying mechanisms of using a combination of KBA and Z-GS as a treatment for ischemic stroke through the application of single-cell transcriptomics. Their analysis revealed that KBA and Z-GS cooperatively modulated the specific gene expression profiles and functionalities within microglia and astrocytes, as well as their subtypes. Furthermore, the key gene responsible for the efficacy of the combination therapy, Spp1, was identified. In conclusion, single-cell transcriptomics provides strong evidence for synergistic treatment of ischemic stroke with KBA and Z-GS.

Network analysis is based on the database to construct network models between drugs, targets and diseases, and to analyze the mechanism of drugs and their impact on diseases. This approach can effectively reveal the synergistic effects of novel combination drugs, assist in multi-target drug development, and provide new perspectives for disease treatment. Jiang et al., 2022 used an integrated strategy combining metabolomics and network analysis to investigate the mechanism of Shentong Zhuyu decoction (STZYD) against rheumatoid arthritis (RA), including *Ligusticum capillaceum H. Wolff* (*Apiaceae*; *“Chuanxiong” Rhizoma*), *Carthamus tinctorius L.* (*Asteraceae; Carthami Flos*), *Prunus persica (L.) Batsch* (*Rosaceae*; *Persicae Semen*), *Achyranthes bidentata Blume* (*Amaranthaceae*; *Achyranthis Bidentatae Radix*), *Gentiana macrophylla Pall.* (*Gentianaceae*; *Gentianae Radix et Rhizoma*), *P. lactiflora Pall.* (*Paeoniaceae*; *Paeoniae Radix Alba*), *Angelica sinensis (Oliv.) Diels (Apiaceae*; *Angelicae Sinensis Radix*), *G. glabra L.* (*Leguminosae; Glycyrrhizae Radix et Rhizoma*), *Notopterygium incisum K.C.Ting ex H.T.Chang* (*Apiaceae*; *Notopterygii Radix et Rhizoma*), *C. myrrha (Nees) Engl.* (*Burseraceae*; *C. myrrha Resina*), *Trogopterus xanthipes Milne-Edwards* (*Muridae*; *Wulingzhi*), *Cyperus rotundus L.* (*Cyperaceae; Cyperi Rhizoma*). Metabolomics of drug-containing serum from rats identified 10 key metabolites, while network analysis was applied to uncover potential targets and pathways against RA. Their results showed that STZYD may exhibit anti-inflammatory effects by acting on the NF-κB signaling pathway, and scientific confirmation of its mechanism of action has been obtained through experiments. Taking everything into account, although network pharmacology holds promise in drug development and elucidating drug mechanisms, it still faces challenges in terms of data quality, biological complexity, and resource requirements. Further validation through experiments is needed.

## 4 Classic cases for AMCT

Novel combination drugs based on AMCT match or exceed the efficacy of TCM formulae, with the added advantage of having clearly defined components. This specificity significantly advances formulae quality control, precise clinical efficacy evaluation and in-depth mechanism of action research, laying a solid foundation for the optimization of treatment methods and clinical application. AMCT’s wide application and significant achievements are demonstrated by classic cases in various disease areas such as cardiovascular diseases, cancer and depression, etc., demonstrating its broad utility and effectiveness.

### 4.1 Cardiovascular disease treatment

Sini decoction (SND), a renowned Chinese medicine formula, is officially recognized in the Chinese Pharmacopoeia as a treatment for cardiovascular syndromes (Wu et al., 1999; Jin et al., 2003). This formula comprises *Aconitum carmichaelii Debeaux* (*Ranunculaceae*; *Aconiti Lateralis Radix Praeparata*), *Zingiber officinale Roscoe* (*Zingiberaceae*; *Zingiberis Rhizoma*), and *G. glabra L.* (*Leguminosae; Glycyrrhizae Radix et Rhizoma*) in a precise mass ratio of 3:2:3 (Yang et al., 2018). Growing research indicates that cardiac mitochondria play a crucial role in the treatment of cardiomyopathy. Specifically, safeguarding against mitochondrial dysfunction in the heart markedly improves treatment results for cardiomyopathy (Szewczyk and Wojtczak, 2002). Ding et al., 2023 employed an advanced 2D CMMC system to identify active metabolites targeting mitochondria within SND. By combining this methodology with the H9c2 cell damage model, they effectively pinpointed three primary metabolites, designated as Songorine (S), Isoliquiritigenin (I), and 8-Gingerol (G). Both *in vitro* and *in vivo* studies have demonstrated that the SGI combination mitigates dilated cardiomyopathy (DCM) through the modulation of mitochondrial energy metabolism and correction of mitochondrial dysfunction, outperforming the effect of any single ingredient used independently. Overall, the SGI combination therapy offers a scientifically supported approach for treating DCM.

Heart failure (HF) is a clinical syndrome characterized by reduced heart contractile and/or diastolic function, where the heart’s blood output fails to meet the body’s metabolic demands. It is a leading cause of death among patients with cardiovascular diseases, signifying a major health concern (Zarrinkoub et al., 2013; Kallikourdis et al., 2017). Liao et al., 2022 employed ultra-high-performance liquid chromatography paired with quadrupole time-of-flight mass spectrometry for the analysis of Si-Miao-Yong-An Decoction (SMYAD) compounds in plasma, including *G. glabra L.* (*Leguminosae; Glycyrrhizae Radix et Rhizoma*), *Lonicera japonica Thunb.* (*Caprifoliaceae*; *Lonicerae Japonicae Flos*), *A. sinensis (Oliv.) Diels (Apiaceae*; *Angelicae Sinensis Radix*), *Scrophularia ningpoensis Hemsl.* (*Scrophulariaceae; Scrophulariae Radix*), a TCM aimed at treating cardiovascular diseases (Zhao et al., 2020; Cui et al., 2021). They successfully identified the main metabolites, angoriside (A) and 3,5-dicaffeoylquinic acid (D). Subsequent experiments revealed that the AD combination reduces myocardial inflammation and enhances heart function by targeting the PDE5A-AKT and TLR4-NOX4 pathways. This intervention decreases ISO-induced excessive autophagic cell death and apoptosis, thereby mitigating and treating heart failure in rat and cellular models induced by ISO.

### 4.2 Tumor treatment

The TCM compound medicine “compound Huang Dai tablet,” a formula used for the treatment of acute promyelocytic leukemia (APL) (Hematol, 2006), includes *Realgar* (As_2_S_2_), *Strobilanthes cusia (Nees) Kuntze* (*Acanthaceae*; *Indigo naturalis*), *Salvia miltiorrhiza Bunge* (*Lamiaceae*; *Salviae Miltiorrhizae Radix et Rhizoma*). Arsenic tetrasulfide (A), Indirubin (I), and Tanshinone IIA (T) are their main effective metabolites. Chen Zhu’s research group showed that the ATI combination yielded enhanced synergy efficacies against APL in murine APL model both *in vivo* and *in vitro*. Mechanistically, ATI potentiated PML-RARα ubiquitination and degradation, enhanced G_1_/G_0_ arrest, promoted the reprogramming of myeloid differentiation regulators, and induced APL cell differentiation, compared with the effects of mono- or biagents (Wang et al., 2008).

Banxia Xiexin Decoction, including *P. ternata (Thunb.) Makino* (*Araceae*; *Pinelliae Rhizoma*), *Coptis chinensis Franch.* (*Ranunculaceae*; *Coptidis Rhizoma*), *Scutellaria baicalensis Georgi* (*Lamiaceae*; *Scutellariae Radix*), *Z. officinale Roscoe* (*Zingiberaceae*; *Zingiberis Rhizoma*), *Panax ginseng C.A.Mey.* (*Araliaceae*; *P. ginseng Radix*), *G. glabra L.* (*Leguminosae; Glycyrrhizae Radix et Rhizoma*), *Ziziphus jujuba Mill.* (*Rhamnaceae*; *Z. jujuba Fructus*), is recognized for its therapeutic applications in treating diseases of the digestive system, including demonstrating efficacy against colorectal cancer (Yan et al., 2019). In a pivotal study conducted by Wang et al., High-Performance Liquid Chromatography (HPLC) was employed to isolate and identify six metabolites with significant presence within the decoction—namely liquiritin, rutin, baicalin, berberine, 6-gingerol, and ginsenoside Rh2. Leveraging the principles of network pharmacology, the study embarked on a comprehensive investigation into the mechanistic pathways through which Banxia Xiexin Decoction, alongside the aforementioned metabolites, exerts anti-colorectal cancer effects. Moreover, experiments using colorectal cancer cell models and subcutaneous tumor-bearing nude mouse models confirmed that the therapeutic effects of a mixture of these six metabolites are comparable to the treatment effects of Banxia Xiexin Decoction itself.

### 4.3 Other disease treatment

The TCM prescription “Yueju,” consisting of *C. rotundus L.* (*Cyperaceae; Cyperi Rhizoma*), *L. capillaceum H. Wolff* (*Apiaceae*; *‘Chuanxiong’ Rhizoma*), *Gardenia jasminoides J.Ellis* (*Rubiaceae*; *Gardeniae Fructus*), *A. lancea (Thunb.) DC.* (*Asteraceae*; *Atractylodis Rhizama*), *Massa Medicata Fermentata*, is a formula used for regulating qi and dispelling melancholy. Gang Chen’s research group showed that this prescription was effective for the treatment of depressive disorder by activating pituitary adenylate cyclase activating polypeptide (PACAP). Geniposide (G) and shanzhiside methyl (S) are the primary metabolites of Yueju based on the HPLC analysis. The GS combination (Geniposide shanzhiside methyl combination dosage following the dosage in the Yueju) enhanced the PACAP-mediated CaMKII/mTOR/BDNF signaling pathway, resulting in a synergistic and prompt antidepressant response (Zhang et al., 2022). Notably, geniposide or shanzhiside methyl did not enhance PACAP signaling and had no significant antidepressant effect when used alone.

Bufei Yishen Formula (BYF), including *Astragalus mongholicus Bunge* (*Fabaceae*; *Astragali Radix*), *P. ginseng C.A.Mey.* (*Araliaceae*; *P. ginseng Radix*), *Adenophora stricta Miq.* (*Campanulaceae*; *Adenophorae Radix*), *Eucommia ulmoides Oliv*. (*Eucommiaceae*; *Eucommiae Cortex*), *Morus alba L.* (*Moraceae*; *Morus Folium et Cortex*), *Rehmannia glutinosa (Gaertn.) DC.* (*Scrophulariaceae*; *Rehmanniae Radix*), *Lycium barbarum L.* (*Solanaceae*; *Lycium Fructus*), *Fritillaria cirrhosa D. Don* (*Liliaceae*; *Fritillariae Cirrhosae Bulbus*), *Aster tataricus L.f.* (*Compositae*; *Asteris Radix et Rhizoma*), *Ophiopogon japonicus (Thunb.) Ker Gawl.* (*Asparagaceae*; *Ophiopogonis Radix*), *A. lancea (Thunb.) DC.* (*Asteraceae*; *Atractylodis Rhizama*), *P. cocos (Schw.) Wolf* (*Polyporaceae*; *Poriae*), has demonstrated significant efficacy in improving clinical symptoms, reducing the frequency of acute exacerbations, and enhancing the quality of life during the stable phase for patients with Chronic Obstructive Pulmonary Disease (COPD) (Li et al., 2012). Li et al., 2020 optimized the combination ratios of 10 metabolites, including ginsenosides, astragalus polysaccharide, astragaloside IV, icariin, schisandrin B, nobiletin, hesperidin, peimine, paeoniflorin, paeonol, in the BYF using orthogonal design, finding the combination ratio to be 22.5:12.5:5:100:12.5:4:30:6.25:2.5:6.25. To verify the efficacy of this novel combination drug compared to the original BYF formula, a rat model of COPD was established using cigarette smoke exposure combined with infection by *Klebsiella pneumoniae*. Treatment outcomes of the novel combination drug and the original BYF formula were observed and compared, showing that the novel combination drug’s efficacy in treating COPD was similar to that of the original BYF formula. Given that the metabolites of the new combination of drugs are more clearly defined, this facilitates the quality control of the formula, the evaluation of clinical efficacy and the study of its mechanism of action, which in turn provides an important basis for the optimization of therapeutic methods and clinical application.

## 5 Challenges and conclusion

Although numerous typical cases have already demonstrated the effectiveness of the AMCT theory, there are still many challenges faced in practice. Firstly, the development and optimization of novel combination drug guided by AMCT theory face significant challenges due to the lack of a standardized evaluation system for identifying and evaluating the active metabolites within these complex metabolites form same TCM formulas. This lack of evaluation system leads to significant variability in the results obtained from different evaluation systems, which complicates the identification of active metabolites. For example, based on target screening or based on the inhibitory effect on a disease model, and how much inhibition is required to be defined as an active metabolite, et al. Secondly, the optimization of the combination ratio of active metabolites is essential for effective disease treatment. Relying solely on traditional experimental methods and disease model such as cell and organoid models, may not fully capture the intricate interactions and synergistic effects among the active metabolites. Such methods might restrict the exploration of combination ratios to a limited range, potentially overlooking more effective or combination ratios that could offer superior therapeutic benefits. Thirdly, the novel combination drugs proposed by the AMCT theory are designed to target specific diseases or classes of diseases, representing a limited scope of indication, as well as novel combination drugs are also influenced by inter-individual differences. Overall, advancing the field of TCM and AMCT necessitates innovations in evaluation methodologies, a deeper understanding of ingredient interactions, and strategies to accommodate individual patient differences, thereby ensuring the development of effective, personalized treatment modalities.

Moreover, the advantages and characteristics of the research and development of Chinese medicine new drugs lie in TCM theories and clinical practice. The TCM prescription, serving as an effective carrier of TCM theories and clinical practice, acts as an important guarantee for the development of novel Chinese medicine. However, the active metabolites and functional mechanisms of the TCM prescriptions are yet elusive, which has restricted the global acceptance of TCM. To promote the development of TCM, a new Chinese medicine research and development model that meets the characteristics of Chinese medicines needs to be established. AMCT rules provide novel thinking for the research and development of novel combination drugs based on clinically effective TCM formulae. Moreover, the integrated use of multi-omics combined analysis, network analysis, artificial intelligence and other cutting-edge technologies are the key to carrying out the research on novel combination drugs novel combination drugs based on clinically effective TCM formulae, which helps to elaborate the mechanism of action of novel combination drugs and improve the applicability of traditional Chinese medicines in the treatment of diseases. AMCT rules may be considered a useful method in exploring the value of TCM prescription on a larger scale, and in promoting the modernization and international needs of TCM.

## Abbreviations

TCM, Traditional Chinese medicine; AMCT, Active metabolites combination therapies; MSI, Mass spectrometry imaging; SPR, Surface plasmon resonance technology; SVM, Random Forests, Support Vector Machines; CI, Synergy index; STZYD, Shentong Zhuyu decoction; TNBC, Triple-negative breast cancer; RA, Rheumatoid arthritis; SND, Sini decoction; HF, Heart failure; SMYAD, Si-Miao-Yong-An Decoction; APL, Acute promyelocytic leukemia; BYF, Bufei Yishen Formula; COPD, Chronic Obstructive Pulmonary Disease.
